# A Phase 1B, randomized, double blind, placebo controlled, multiple-dose escalation study of NSI-189 phosphate, a neurogenic compound, in depressed patients

**DOI:** 10.1038/mp.2015.178

**Published:** 2015-12-08

**Authors:** M Fava, K Johe, L Ereshefsky, L G Gertsik, B A English, J A Bilello, L M Thurmond, J Johnstone, B C Dickerson, N Makris, B B Hoeppner, M Flynn, D Mischoulon, G Kinrys, M P Freeman

**Affiliations:** 1 Massachusetts General Hospital, Clinical Trials and Network Institute, Boston, MA, USA; 2 Harvard Medical School, Department of Psychiatry, Boston, MA, USA; 3 Neuralstem Inc., Germantown, MD, USA; 4 PAREXEL International, Los Angeles Early Phase Unit, Los Angeles, CA, USA; 5 California Clinical Trials Medical Group, Glendale, CA, USA; 6 Ridge Diagnostics, Research Triangle Park, NC, USA; 7 Q-Metrx, Inc., Glendale, CA, USA; 8 Center for Morphometric Analysis, Massachusetts General Hospital, Boston, MA, USA

## Abstract

We wanted to examine tolerability and efficacy of NSI-189, a benzylpiperizine-aminiopyridine neurogenic compound for treating major depressive disorder (MDD). This was a Phase 1B, double blind, randomized, placebo controlled, multiple-dose study with three cohorts. The first cohort received 40 mg q.d. (*n*=6) or placebo (*n*=2), the second cohort 40 mg b.i.d. (*n*=6) or placebo (*n*=2), and the third cohort 40 mg t.i.d. (*n*=6) or placebo (*n*=2). Twenty-four patients with MDD were recruited, with the diagnosis and severity confirmed through remote interviews. Eligible patients received NSI-189 or placebo for 28 days in an inpatient setting with assessments for safety, pharmacokinetics (PK) and efficacy. Outpatient follow-up visits were conducted until day 84 (±3). NSI-189 was relatively well tolerated at all doses, with no serious adverse effects. NSI-189 area under the curve increased in a dose-related and nearly proportional manner across the three cohorts, with a half-life of 17.4–20.5 h. The exploratory efficacy measurements, including Symptoms Of Depression Questionnaire (SDQ), Montgomery-Asberg Depression Scale (MADRS), Clinical Global Impressions—Improvement (CGI-I), and The Massachusetts General Hospital (MGH) Cognitive and Physical Functioning Questionnaire (CPFQ) showed a promising reduction in depressive and cognitive symptoms across all measures for NSI-189, with significant improvement in the SDQ and CPFQ, and a medium to large effect size for all measures. These improvements persisted during the follow-up phase. In summary, NSI-189 shows potential as a treatment for MDD in an early phase study. The main limitation of this preliminary study was the small sample size of each cohort.

## Introduction

Despite the number of pharmacologic agents on the market for the treatment of depression, novel antidepressants with efficacy and tolerability are still in much need. Only approximately one-third of patients with major depressive disorder (MDD) achieve full remission of symptoms with currently available antidepressants.^1, 2, 3, 4^ Currently available antidepressants generally share a mechanism of action focused on the monoamine neurotransmitter system, suggesting that the most robust potential advances in the field may be most achievable by the utilization of mechanisms of action different from those of currently available medications.^5^ Importantly, currently available antidepressants are associated with adverse effects that may negatively impact treatment adherence, such as gastrointestinal symptoms, agitation, sleep and weight changes, and sexual dysfunction.^6^

Neurogenesis in the hippocampal formation has been demonstrated throughout the lifetime of multiple species including humans.^7^ Treatment with antidepressant drugs increases hippocampal neurogenesis, and it has been hypothesized that increasing adult hippocampal neurogenesis may be a new drug target or mechanism for future antidepressant drugs.^7, 8^ Therefore, an accumulating body of evidence strongly supports the hypothesis that a platform of pre-clinical neurogenesis paired with confirmatory behavioral assays may be useful as a drug discovery strategy.

NSI-189, a benzylpiperizine-aminiopyridine, is a novel chemical entity that stimulates neurogenesis of human hippocampus-derived neural stem cells *in vitro* and stimulates neurogenesis in mouse hippocampus *in vivo* (Data on file, Neuralstem, non-peer-reviewed). It has shown behavioral efficacy at three different doses in a mouse model of depression (novelty suppressed feeding) after daily oral administration for 28 days (Data on file, Neuralstem, non-peer-reviewed). The brains of treated mice showed significantly increased neurogenesis in the dentate gyrus and significantly increased hippocampal volume (Data on file, Neuralstem, non-peer-reviewed). NSI-189 is believed to have a highly specific effect in the hippocampus and subventricular zone, the two well-known neurogenic regions in adult central nervous system, and nowhere else in the central nervous system (Data on file, Neuralstem, non-peer-reviewed). *In vitro* studies with NSI-189 showed limited metabolism involving oxidative processes combined with some evidence for glucuronide conjugation (Data on file, Neuralstem, non-peer-reviewed). Estimates of total clearance of NSI-189 (CL/F) in healthy normal subjects indicated moderate clearance approximating hepatic blood flow (Data on file, Neuralstem, non-peer-reviewed). Urine samples from preclinical or clinical testing have not been analyzed.

This double blind, randomized, placebo controlled, multiple-ascending dose study with three cohorts was designed to primarily address safety and PK objectives in MDD patients. The primary goal of the study was to identify the maximum safe dose of NSI-189 that could be administered for at least 28 days in MDD patients and to determine multiple dose/steady-state PK. Second, exploratory analyses of its effects on depressive symptoms and quantitative electroencephalogram (qEEG) measures were conducted, as well as hippocampal volumetric changes.

## Materials and methods

This was a double blind, randomized, placebo controlled, multiple-dose study to assess the safety and PK of NSI-189 phosphate in patients with MDD. The study was performed in male and female patients 18–60 years of age, inclusive, diagnosed with MDD, recurrent, as per the Diagnostic and Statistical Manual of Mental Disorders (DSM-IV-TR) criteria and confirmed by the Structured Clinical Interview for DSM-IV specific for Clinical Trials (SCID-CT). Staff psychiatrists/psychologists at the MGH Clinical Trials Network and Institute performed independent remote SAFER (state versus trait; assessability; face validity; ecological validity; and rule of three Ps (pervasive, persistent, and pathological)) interviews^9, 10^ for screened patients who the sites deemed eligible for randomization to confirm validity of the diagnosis of MDD and eligibility for the study. The study implementation, including magnetic resonance imaging (MRI) and qEEG assessments, and patient recruitment was conducted at the Parexel Los Angeles Early Phase Clinical Unit. After complete description of the study to the subjects, written informed consent was obtained by each participant. Patients at screening were either medication free with a history of taking antidepressant medication(s) in the past for their depressive disorder, or were allowed to be on antidepressant medication(s). Eligible patients on antidepressants were admitted to the unit on day −5 to complete a wash-out period, and were reconfirmed for eligibility at the baseline assessments. Eligible patients not on antidepressant medication when enrolled in the study were admitted to the unit on day −2 for reconfirmation of eligibility and baseline assessments.

**Figure 1:**
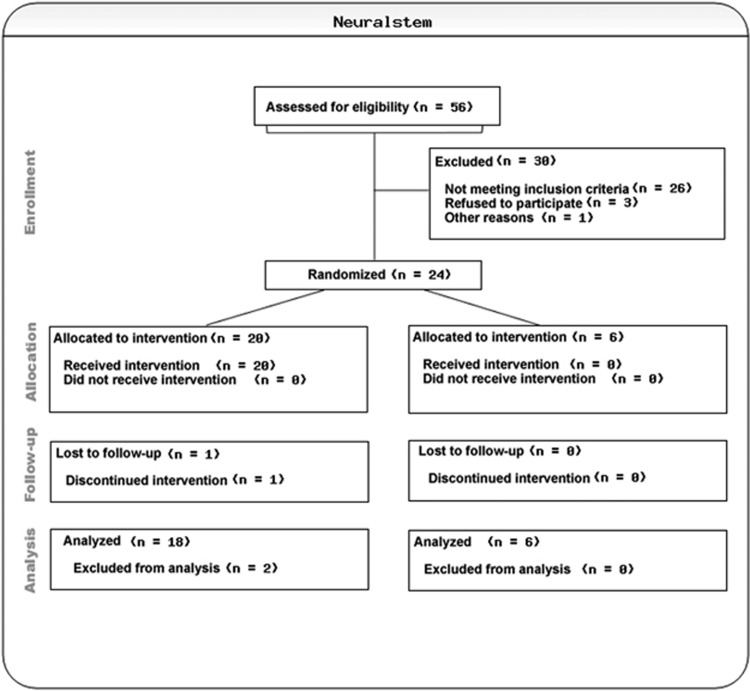
Consort chart.

Twenty-four patients were planned for enrollment into three dose groups (40 mg q.d., 40 mg b.i.d., and 40 mg t.i.d.) with eight patients per cohort. Within each cohort (with at least three female patients), six patients were randomized to NSI 189 phosphate and two patients were randomized to placebo, in this double blind, randomized, placebo controlled, multiple-dose study with three ascending cohorts (*N*=24) (Figure 1).

The randomization code was provided by Max Neeman International, India, before the study. Two sets of sealed code break envelopes were supplied. One set was kept at the Clinical Unit and the other set was sent to the PAREXEL Pharmacovigilance reporting department. Dose escalation by titrating from 40 mg q.d. to 40 mg b.i.d. and 40 mg t.i.d. drug administration was based on pre-clinical findings demonstrating central nervous system-related adverse events in animals associated with *C*_max_ (Data on File, Neuralstem). Prior Phase 1 clinical studies with doses of NSI-189 up to 80 mg as a single dose demonstrated no drug-related adverse events (Data on File, Neuralstem). Therefore, to reduce the risk of possible peak-related adverse events, split daily dosing was used to permit higher exposures of NSI-189 without increasing the risk of peak-related adverse events (AEs). During the study, patients underwent a screening visit (day −37 to −6), Admission and washout (day −5 to −1) for patients treated with antidepressant medication(s) at screening and (day −2 to −1) for patients not being treated with antidepressant medication(s) at screening, in-house double-blind dosing (day 1 to 28), and follow up and end-of-study visits (day 35 (±3), day 42 (±30), day 49 (±3), day 70 (±3), day 56 (±3) and day 84 (±3) (end of study)). During the in-house dosing, safety, PK and PD assessments took place relative to dosing. In addition to having the study IRB approved, a Safety Monitoring Board reviewed 7-day safety data of all patients within each dose cohort, before proceeding to the next ascending dose cohort, according to the safety stopping criteria outlined in the protocol. A total of eight patients were on psychotropic medications requiring tapering off before randomization per protocol. A total of six patients were restarted on psychotropic medications post day 28. There were two patients who were initiated on different antidepressants than the ones they originally were titrated off.

### Inclusion criteria

Patients who met the following criteria were considered eligible to participate in the study:

1. Males and females 18–60 years of age, inclusive.
2. Diagnosis of MDD, recurrent, as per the DSM-IV-TR criteria and confirmed by the Structured Clinical Interview-Clinical Trials. Their major depressive episode was confirmed via SAFER interview^9^ administered by remote, independent raters.
3. MADRS^11^ score of 15–30, inclusive, at screening and baseline, confirmed via independent interview administered by remote, independent raters.
4. The following applied to female patients only: non-pregnant, non-lactating females of childbearing potential who agreed to use medically acceptable forms of birth control from screening until the end of study.
5. The following applied to male subjects: male subjects with a female partner of childbearing potential were required to use an effective method of birth control or practice abstinence during this study and for 3 months following discontinuation of study medication.
6. Body mass index of ⩾19.5 and ⩽35.0 kg/m^2^ at screening. Bodyweight must be >50 kg.

### Exclusion criteria

Patients meeting one or more of the following criteria were not considered eligible to participate:

1. Clinically significant history or evidence of cardiovascular, respiratory, hepatic, renal, gastrointestinal, endocrine, neurological, immunological, dermatological or psychiatric disorder(s), including substance abuse or dependence, or other major disease.
2. Clinically significant suicidal or homicidal ideation or behavior currently or during the preceding 6 months.
3. Clinically significant abnormal clinical laboratory tests or ECGs.
4. Regular use of any tobacco product exceeding 10 cigarettes or their equivalent within 3 months before day 1.

### Efficacy measures

The following measures were administered with both clinicians and patients being blind to treatment assignment:

1. Montgomery-Asberg Depression Rating Scale (MADRS):^11^ this 10-item clinician-rated instrument measures depression severity.
2. CGI-S and Improvement (CGI-I) Scales.^12^ These clinician-rated scales rate the severity of the disorder and the global improvement as the beginning of the study.
3. SDQ:^13^ This validated self-rating instrument has 44 items on a scale of 1–6, measuring multiple depressive symptom domains.
4. MGH CPFQ:^14^ this is a brief (7-item), validated self-report inventory to assess rates of significant cognitive symptoms such as memory, attention and executive functioning difficulties.

### Hippocampal and amygdala volumes

Structural MRI scans were acquired using a GE Signa, 1.5 T Excite HD MRI (GE Medical Systems, Milwaukee, WI, USA) with a 16-channel head coil. During scanning, the participant's head was immobilized using tight foam padding to minimize motion. Each participant underwent sagittal T1-weighted, three-dimensional, non-contrast enhanced, inversion recovery high-resolution spiral imaging (IR-SPGR) sequence, with the following acquisition parameters: 1-mm-isotropic voxel size, inversion time 500 ms, repetition time 8.5 ms, echo time 3.75 ms, flip angle 10 deg and field of view 192 × 192 mm, slice thickness 1.2 mm, number of slices 170. Four scans were obtained for each subject at baseline, day 28, 56 and 84. MRI data were processed using the standard longitudinal processing stream in FreeSurfer 5.3 (Massachusetts General Hospital, Boston, MA, USA).^15^ The automated segmentation procedures were used to segment the left and right hippocampal and amygdala formation for each subject at each time point. Each subject's scan and segmentation were visually inspected by a trained operator to confirm accuracy according to a previously published protocol.^16^ No manual edits were performed. Hippocampal and amygdala volume measurements were obtained for each subject at each time point (in mm^3^). Because of technical problems with scan acquisitions, data were not available for *n*=3 at day 84 (two placebo, one NSI-189).

### Peripheral biomarker and pharmacokinetic assessments

#### Pharmacokinetics

Per protocol, plasma concentrations of NSI-189 were collected at specified time points relative to NSI-189 dosing at day 1 up to day 84. Blood samples for the determination of the plasma concentrations of NSI-189 were collected before dose administration (predose) on day 1 and then throughout the 31 days, and 3 days after the last dose on day 28. Full profiles were measured on day 1, after either 1, 2, or 3 doses, on day 14, and after the last dose on day 28. Samples were measured using a validated LC/MS/MS method by WIL Research (Ashland, OH, USA). Pharmacokinetic parameters for NSI-189 in plasma were calculated using non-compartmental analysis using individual patient concentrations vs time graphs using SAS for Windows (Version 9.3, SAS Institute, Cary, NC, USA). The lower limit of quantitation for NSI-189 in human plasma was 1 ng ml^−1^. In this study, PK was not evaluated on fasted subjects. In the previous, Phase 1a study, PK was done after overnight fasting and, in a substudy, we compared PK of fasted vs fed. Food did not affect the overall drug exposure or the Cmax (Data on File, Neuralstem). Therefore, in the current study, PK was conducted without overnight fasting. Subjects were dosed 1–4 h before meal and no food was provided for 2 h post dose.

#### Peripheral biomarkers

Each subject had a blood sample drawn at the indicated time points ranging from −5 to 84 days post treatment. Plasma was stored frozen at ⩽−70 °C until shipment to Ridge Diagnostics (Research Triangle Park, NC, USA) for analysis. Plasma samples were tested by immunoassay for plasma levels of 10 biomarkers (alpha-1-antitrypsin (A1AT), apolipoprotein C3 (ApoC3), brain-derived neurotrophic factor, cortisol, epidermal growth factor, myeloperoxidase, prolactin, resistin, soluble tumor necrosis factor receptor type 2, and thyroid stimulating hormone). The majority of the analytes were measured by enzyme-linked immunosorbent assay, whereas A1AT and apolipoprotein C3 were measured by turbidimetric assays developed by Ridge. There was no difference in the frequency of blood draws between placebo and active subjects. The site staff was blinded to treatment nature. However, the peripheral biomarker analysis was carried out in frozen samples once the study was completed and the study was unblinded. Therefore, biomarker levels of each analyte were measured at specified intervals for each subject from day 1 through day 84 treated with NSI-189 (*n*=18) for 28 days. Biomarker levels in patients who received placebo (*n*=6) were instead measured only twice (baseline and day 28) during that time period.

#### Safety and qEEG

Safety EEG assessments were obtained for all cohorts at screening visit, day 1, 14 and 28, at 1 h and 3 h post dose, bracketing the *T*_max_ of NSI-189 and were relative to the dosing frequency (that is, 40mg q.d. after first dose, every morning; 40 mg b.i.d. after 2nd dose, early evening; and 40 mg t.i.d. after the third dose of the day, after midnight). In addition safety EEG assessments were performed at days 3 and 7 for the 40 mg b.i.d. and 40 mg t.i.d. cohorts only. Digital EEGs (Cadwell Laboratories, Kennewick, WA, USA) were recorded with subjects resting with eyes closed using 19 scalp electrodes placed according to the International 10/20 System, and including ECG, and eye movement monitoring. Further, EEG recordings obtained at the baseline visit and 6 h post-dose on day 14 and 28 were analyzed using power spectral analysis. Physiological and instrumentation artifacts were identified and removed from EEG files manually by an experienced technologist. Epochs of cleaned EEG data are submitted to power spectral analyses using Brain Vision Analyzer software (Brain Products, Gilching, Germany) and results were banded over the 4–6, 6–8, 8–10 and 10–12 Hz frequency ranges for each recording electrode.

### Statistical analyses

Differences across cohorts in clinical and demographic characteristics were analyzed using *χ*^2^ test for categorical variables and analysis of variance for continuous variables. Spontaneous adverse events were descriptively tabulated. With respect to the exploratory analyses of efficacy, the three cohorts were combined to increase power so that the placebo group had six subjects, and the NSI-189 group (all doses) had 18 subjects. Analysis of covariance was used for the following dependent variables: CGI-I, MADRS, SDQ, CPFQ and hippocampal and amygdala volumes observed at day 28 (end of treatment) and day 84 (end of follow-up). Covariates were the baseline values to adjust for initial differences (for CGI-I, day 7 was used as covariate). Excluding *n*=2 early termination patients (replaced) and incomplete follow-up (that is, missing day 84) for *n*=2 (not replaced). Effect sizes were estimated using Cohen's^17^ *d* group mean differences: *d*=0.20 (small); *d*=0.50 (medium); *d*=0.80 (large). A *post hoc* analysis was conducted on the hippocampal and amygdala volume data, as data were missing for two out of six placebo patients at day 84. Thus, a repeated measures analysis of variance (ANOVA) was conducted in the pooled NSI-189 group only to test if there was a trend over time. Data analyses for qEEG measures were based on repeated-measures analysis of variance and Student's *t* as appropriate. Logistic regressions were carried out to analyze the peripheral biomarkers.

## Results

The study successfully recruited 24 MDD patients. Table 1 summarizes the clinical and demographic characteristics of the sample. The patients assigned to placebo in the three cohorts were numerically younger than patients assigned to active treatment. Among the pooled active group patients (*n*=18), seven (38.9%) were Caucasian, seven (38.9%) were African-American, three (16.7%) were Hispanic and one (5.6%) was Asian, compared with the pooled placebo group, where four (66.6%) were Caucasian, zero (0%) were African-American, 2 (33.3%) were Hispanic and zero (0%) were Asian. With respect to gender, seven (38.9%) of the pooled active group patients (*n*=18) were males vs seven (83.3%) of the pooled placebo group (*n*=6). Two patients dropped out of the study post randomization, and both were replaced. The first patient was screened on 24 May 2012, and the first dosing was 12 June 2012. The last subject, last visit was on 13 November 2013 (Table 1).

**Figure 2:**
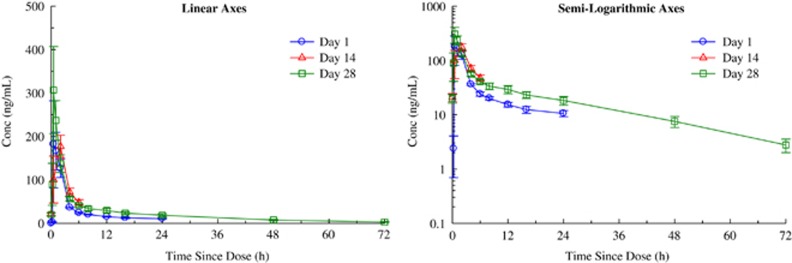
Mean±s.e. plasma concentrations of NSI-189 on days 1, 14 and 28 during oral administration of 40 mg Q24H to subjects with MDD—linear axes (left panel) and semi-logarithmic axes (right panel). MDD, major depressive disorder.

**Figure 3:**
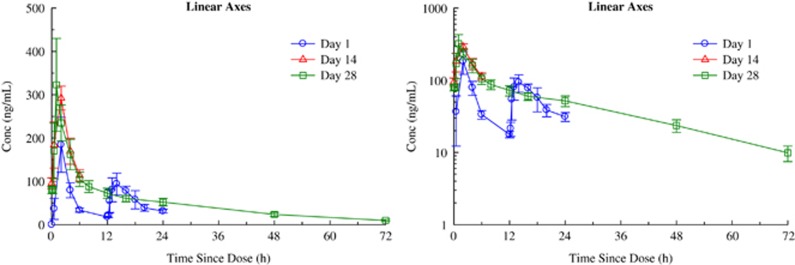
Mean±s.e. plasma concentrations of NSI-189 on days 1, 14, and 28 during oral administration of 40 mg Q12H to subjects with MDD—linear axes (left panel) and semi-logarithmic axes (right panel). MDD, major depressive disorder.

**Figure 4:**
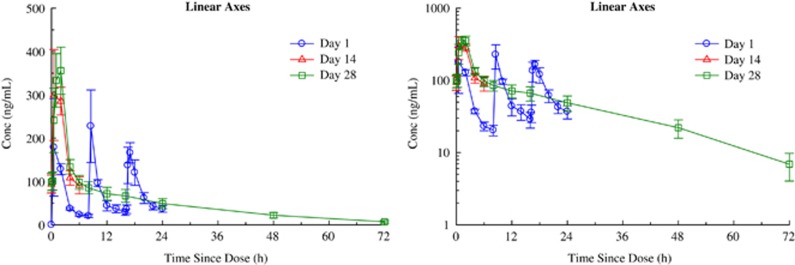
Mean±s.e. plasma concentrations of NSI-189 on days 1, 14, and 28 during oral administration of 40 mg Q8H to subjects with stable depression — linear axes (left panel) and semi-logarithmic axes (right panel). MDD, major depressive disorder.

With respect to the primary aim of the study, NSI-189 was well tolerated supporting the dose escalation up to 40 mg t.i.d. by the Safety Monitoring Board. Table 2 summarizes the spontaneously reported adverse events. Administration of 40 mg doses of NSI-189 q.d., 40 mg b.i.d. and 40 mg t.i.d. for 28 days resulted in accumulation consistent with the mean *T*_½_ of 17.4±3.06, 20.5±3.51 and 18.6±4.14 h, respectively. Steady state was reached after 96–120 h, consistent with the *T*_½_ and independent of the dosing regimen. Figures 2, 3, 4 provide the pharmacokinetics curves obtained with the study. The predicted accumulation for the q.d. regimen was in good agreement with the observed values based on *C*_max_ and area under the curve (AUC) (0–*τ*), whereas those for the b.i.d. and t.i.d. regimens were roughly proportional. Day 28 AUCs (0–24) were 1144±276, 2791±1443 and 4384±1217 h × ng ml^−1^ for the q.d., b.i.d. and t.i.d. regimens, respectively. Mean time to maximal peak concentration (*T*_max_) ranged from 1–2 h at day 1 and 28. There were no obvious pharmacokinetic differences between males and females (Table 2).

### Efficacy

**Figure 5:**
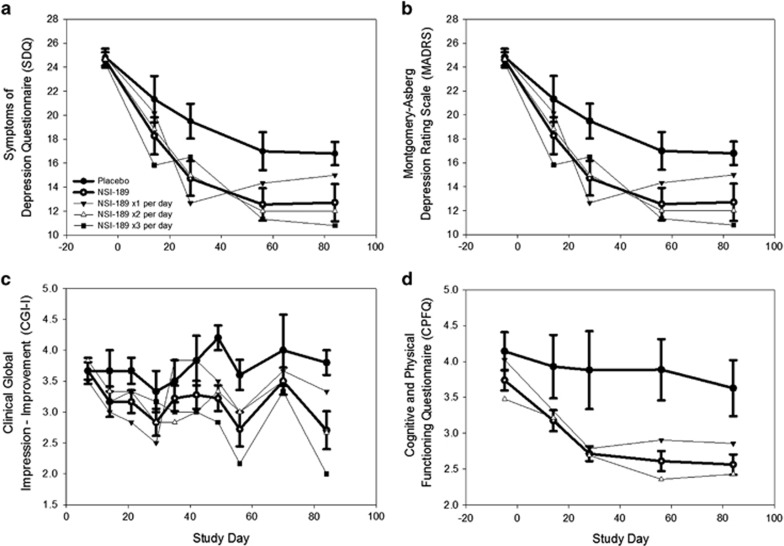
(**a**–**d**) Efficacy outcomes in means over time.

With respect to the exploratory efficacy assessments, Figure 5a shows the results of the analyses of the SDQ. There was a significantly greater reduction in depressive symptoms at day 28 (the end of the double-blind phase) in the pooled NSI-189 patients compared with the placebo-treated patients on the SDQ. The significant advantage of the pooled NSI-189 patients compared with the placebo-treated patients was maintained through the end of the follow-up period (day 84). Figures 5b and c show trends toward significantly greater reduction in depressive symptoms as measured by the MADRS and the CGI-I at day 28. These trends were maintained through the end of the follow-up period (day 84), although the differences between drug and placebo were not statistically significant. These treatment responses were for the most part in patients who did not restart prior treatments, if they were on any. For the remainder of the patients, the treatments that were restarted were considered not or only partially effective prestudy. Figure 5d demonstrates the significant reduction in symptoms on the CPFQ at day 28 in the pooled NSI-189 patients compared with the placebo-treated patients, maintained until the end of the follow-up period. The effect sizes detected with the four depression outcome measures ranged from 0.57 (CGI-I) to 0.90 (SDQ), 0.94 (CPFQ), and 0.95 (MADRS), consistent with a medium to large effect size (Figures 5a–d).

### Hippocampal and amygdala volumes

**Figure 6:**
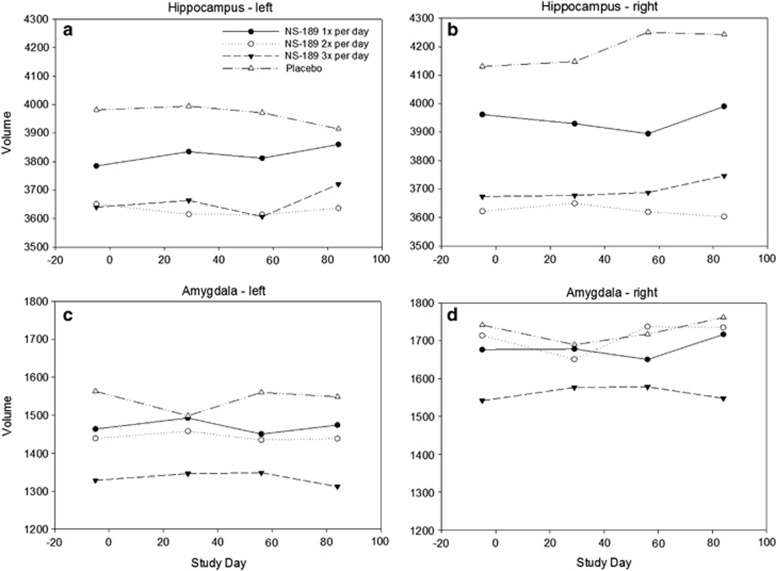
The means over time of the hippocampal and amygdala volumes (**a**–**d**).

Figures 6a–d describe the means over time of the hippocampal and amygdala volumes. Analysis of covariance results did not show significant differences in hippocampal volume at day 28 (left: *F*(1,21)=0.06, *P*=0.80; right: *F*(1,21)=0.71, *P*=0.41) or day 84 (left: *F*(1,18)=1.25, *P*=0.28; right: *F*(1,18)=4.17, *P*=0.06) between the pooled NSI-189 and placebo-treated patients. Results were also non-significant for the control site, the amygdala, at day 28 (left: *F*(1,21)=4.30, *P*=0.05; right: *F*(1,21)=0.63, *P*=0.44) or day 84 (left: *F*(1,18)=0.00, *P*=0.98; right: *F*(1,18)=0.01, *P*=0.91). The *post hoc* repeated measures analysis of variance suggested a modest but not statistically significant increase in the left hippocampal volume in the NSI-189-treated patients (*b*=0.35, *P*=0.12), but not the right side (right: *b*=−0.03, *P*=0.82). In the control site, the trend was non-significant on the left side (*b*=−0.03, *P*=0.74), and significant on the right side (*b*=0.32, *P*=0.049).

### Peripheral biomarker assessments

Plasma levels of a subset of biomarkers at baseline did partially predict the clinical outcome after 28 days of treatment. Of the 18 active patients who completed the study, 2 were non-responders in that their MADRS score remained within the original moderate depression classification (MADRS score 20–34). Twelve^13^ treated patients were designated responders, defined as those who had either 50% drop in MADRS score or returned to a normal range (MADRS score 0–6). Four (4) patients were designated partial responders since the post-treatment MADRS score indicated a change from moderate to mild depression. We used logistic regression modeling of baseline biomarker data in this limited sample to predict whether an individual patient fully or partially responded to treatment with NSI-189. In this analysis, we used the MADRS scores at day 28 the last treatment day. Using baseline plasma levels of brain-derived neurotrophic factor, epidermal growth factor, myeloperoxidase, tumor necrosis factor receptor type 2 and A1AT, the model, with one exception (patient 608), was able to identify the response to NSI-189, in 17 out of 18 cases that are consistent with therapeutic effects shown by the decrement in MADRS score. Table 3 indicates the individual patient identifier, and the response group designation (R, responder; PR, partial responder; NR, non-responder) and the predicted probability of response based on analysis of baseline biomarker data.

### Safety and qEEG

**Figure 7:**
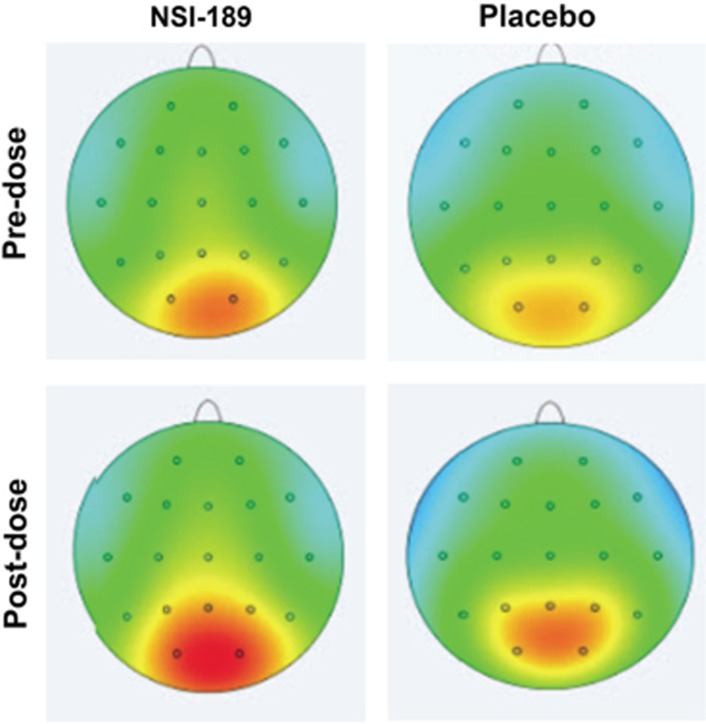
Topographs of high-frequency alpha (10–12 Hz) comparing baseline with day 28.

Results of safety EEG assessments bracketing the *T*_max_ (1–2 h) of NSI-189 revealed no seizure activity in any case. No new EEG findings were identified by visual inspection post dose that were persistent in final recordings. One subject showed sharp activity that was not seen in the recording at discharge. Results of qEEG analyses show increased high-frequency alpha with active treatment and lower high-frequency alpha, or less change with placebo. Figure 7 displays the topographs of high-frequency alpha (10–12 Hz) comparing baseline with day 28. This effect is particularly prominent in the left posterior temporal (T5; *t*=2.45, *P*=0.0247) and left parietal regions (P3; *t*=3.31, *P*=0.004) in the active treatment group and is similar when comparing quantitative EEG at baseline to either day 14 or 28 assessments. Significant univariate effects comparing amplitude from baseline to post-dose assessments are seen only for changes within the active treatment group.

## Discussion

The primary aims of this study concerning the analyses of the safety, tolerability and pharmacokinetics of NSI-189 were achieved. In this Phase 1b study, NSI-189, a novel chemical entity that stimulated neurogenesis in earlier studies, was well tolerated, and administration of 40 mg doses of NSI-189 Q24H, Q12H and Q8H for 28 days resulted in accumulation consistent with the mean *t*_½_ of 17.4–20.5 h, with steady state being reached after 96–120 h, consistent with the *t*_½_ and independent of the dosing regimen. The study also showed that NSI-189 had significantly greater antidepressant effects than placebo in two (SDQ and CPFQ) of the four depression outcome measures. Although statistical significance was achieved only with the self-rating scales, presumably because of the tighter s.d., the effect size detected with the clinician-rated MADRS (0.95) was similar to the effect sizes of the two self-rated depression outcome measures (0.90 for the SDQ and 0.94 for the CPFQ). Plasma levels of a subset of biomarkers at baseline did partially predict the clinical outcome after 28 days of treatment. MRI data suggested a potential modest increase in hippocampal volume in NSI-189-treated patients, which would be in line with the postulated neurogenetic mechanism underlying NSI-189 treatment, but this was not significant and was not different from the changes observed on the small cohort on placebo, and are purely provided for descriptive purposes. Also note that trends of similar magnitude were observed in the control site, the amygdala, thereby underscoring the possibility of a spurious finding.

The study results are consistent with the growing body of evidence that treatment with antidepressant drugs increases hippocampal neurogenesis and with the hypothesis that pre-clinical neurogenesis matched with confirmatory behavioral assays may be useful as a drug discovery strategy.^7, 8^

It is worth noting that the significant benefits on the SDQ and the trends for improvement on the MADRS and CGI-I were maintained steadily beyond the acute phase of the double-blind administration and were still present at day 84, with the exception perhaps of the 40 mg q.d. regimen. This finding is in sharp contrast to the rapid return of symptoms typically observed following discontinuation of standard antidepressants (with relapse rates hovering ~20% on placebo 2 months after antidepressant treatment discontinuation).^18^ These treatment responses were for the most part in patients who did not restart prior treatments, if they were on any. For the remainder of the patients, the treatments that were restarted were considered not or only partially effective prestudy. Biomarker changes were not collected in the post-treatment period. If replicated in a future study, this observation suggests the presence of unique features of NSI-189, such as a more durable antidepressant effect.

Another interesting finding related to NSI-189 is the significant improvement compared with placebo in cognitive symptoms, as measured by the CPFQ. Future studies should explore whether such pro-cognitive effect may be all explained by the improvement in depressive symptoms, or instead by specific effects of the increased neurogenesis on cognition, given the established relationship between these two.^19^ Exploratory analyses of qEEG changes demonstrated significant changes indicating a direct influence on the nervous system, and these changes were seen only for the active NSI-189 group. Interestingly these findings are best seen in regions near the hippocampus, and were more prominent on the left. Neuropsychological correlates of these changes would be expected to include modulating context regulation of affect.^20^

Preliminary analysis of a small sample set with a low ‘event per variable' ratio suggests that prediction of response by analyzing peripheral biomarkers may be possible from baseline biomarker profiling in plasma samples. In that regression model brain-derived neurotrophic factor, epidermal growth factor, myeloperoxidase, tumor necrosis factor receptor type 2 and A1AT are key analytes.

The main limitation of the study is the small sample size (*n*=24) and the unbalanced randomization, with 24 subjects assigned to active treatment and only 6 to placebo, although this is a design typical of Phase 1 studies. These two issues may increase the likelihood of false positives. In addition patients who were willing to participate in this study agreed to an inpatient stay, which may not be representative of patients with MDD. Despite these limitations, each cohort seemed to have consistently shown an antidepressant effect and the overall effect sizes were quite robust. MDD treatment trials often have high placebo response rates, and the opportunity for signal detection in a small early phase study is usually quite limited.

However, this Phase 1b study included a series of methodological innovations which may have contributed to the success of the study. Staff psychiatrists/psychologists at the MGH Clinical Trial Network Institute performed independent remote SAFER interviews^9^ for screened patients the sites deemed eligible for randomization to assess validity of a diagnosis of depression and eligibility for the study. Therefore, both diagnosis and appropriate illness severity were independently verified. This verification may have contributed to a relatively low placebo response rate, as shown in previous studies of our group.^21, 22^ Another innovation involves the use of a relatively more comprehensive measure of depression, the self-rated SDQ, a valid and reliable questionnaire assessing psychological, physical and behavioral symptoms of depression.^13^ This instrument is more in line with the Research Domain Criteria approach to assessment of depressed patients^23^ and includes measures of irritability, anger attacks and anxiety symptoms together with the commonly considered symptoms of depression.

In summary, consistent with findings from our group that a neurogenic combination therapy was more effective than placebo in MDD,^24^ a novel neurogenic compound, NSI-189, has shown promise as a potential treatment for MDD in a Phase 1B, double-blind, randomized, placebo controlled, multiple-dose study with three ascending cohorts.

## Figures and Tables

**Table 1:** Patients' demographics and clinical characteristics

	*Pooled placebo (*n=*6)* N *(%)*	*40 mg q.d. (*n=*6)* N *(%)*	*40 mg b.i.d. (*n=*6)* N *(%)*	*40 mg t.i.d. (*n=*6)* N *(%)*	P
*Ethnic origin, n (%)*					
Caucasian	4 (66.6)	3 (50.0)	2 (33.3)	2 (33.3)	0.604^a^
African American	0 (0.0)	2 (33.3)	3 (50.0)	2 (33.3)	
Hispanic	2 (33.3)	1 (16.6)	1 (16.6)	1 (16.6)	
Asian	0 (0.0)	0 (0.0)	0 (0.0)	1 (16.6)	
Gender, *n* (%)					0.262^a^
Male	5 (83.3)	2 (33.3)	3 (50.0)	2 (33.3)	
Female	1 (16.6)	4 (66.6)	3 (50.0)	4 (66.6)	
Age (year), mean (s.d.)	28.20 (4.75)	34.00 (3.50)	38.50 (12.94)	40.50 (9.92)	0.098^b^
MADRS total score at screening, mean (s.d.)	25.20 (2.93)	26.00 (3.35)	26.83 (2.64)	24.83 (2.56)	0.636^b^

Abbreviation: ANOVA, analysis of variance.

a Statistical method used, *χ*^2^ test.

b Statistical method used, one-way ANOVA.

**Table 2:** Adverse events^a^

*Side effect*	*Pooled placebo*	*40 mg q.d.*	*40 mg b.i.d.*	*40 mg t.i.d.*	*Pooled active*	*Pooled placebo*
	(n=*6)* N *(%)*	(n=*6)* N *(%)*	(n=*6) N (%)*	(n=*6) N (%)*	(n=*18)* N *(%)*	(n=*6)* N *(%)*
*Autonomic*						
Dry mouth	0 (0%)	0 (0%)	2 (33.3%)	0 (0%)	2 (11.1%)	–
Palpitation	0 (0%)	1 (16.7%)	0 (0%)	0 (0%)	1 (5.6%)	–
						
*CNS/psychiatric*						
Headache	3 (50.0%)	3 (50.0%)	3 (50.0%)	3 (50.0%)	9 (50%)	3 (50%)
Dizziness	1 (16.7%)	0 (0%)	1 (16.7%)	4 (66.7%)	5 (27.8%)	1 (16.7%)
Somnolence	1 (16.7%)	3 (50.0%)	1 (16.7%)	1 (16.7%)	5 (27.8%)	1 (16.7%)
Fatigue	0 (0%)	1 (16.7%)	0 (0%)	0 (0%)	1 (5.6%)	–
Restlessness	0 (0%)	0 (0%)	0 (0%)	1 (16.7%)	1 (5.6%)	–
Poor quality of sleep	0 (0%)	1 (16.7%)	0 (0%)	0 (0%)	1 (5.6%)	–
Nightmare/vivid dream	0 (0%)	1 (16.7%)	1 (16.7%)	1 (16.7%)	3 (16.7%)	–
Paresthesia	0 (0%)	1 (16.7%)	0 (0%)	1 (16.7%)	2 (11.1%)	–
Insomnia	0 (0%)	1 (16.7%)	1 (16.7%)	1 (16.7%)	3 (16.7%)	–
Irritability	0 (0%)	1 (16.7%)	0 (0%)	0 (0%)	1 (5.6%)	–
Difficulty concentrating	1 (16.7%)	0 (0%)	0 (0%)	0 (0%)	–	1 (16.7%)
Hyperthymia	1 (16.7%)	0 (0%)	0 (0%)	0 (0%)	–	1 (16.7%)
						
*Gastrointestinal*						
Dyspepsia	1 (16.7%)	0 (0%)	0 (0%)	0 (0%)	–	1 (16.7%)
Abdominal pain	1 (16.7%)	0 (0%)	0 (0%)	0 (0%)	–	1 (16.7%)
Nausea	0 (0%)	0 (0%)	0 (0%)	2 (33.3%)	2 (11.1%)	–
						
*Skin and subcutaneous tissue disorders*						
Skin pain	0 (0%)	1 (16.7%)	0 (0%)	0 (0%)	1 (5.6%)	-
Rash	0 (0%)	0 (0%)	0 (0%)	1 (16.7%)	1 (5.6%)	-

Abbreviations: AE, adverse events; CNS, central nervous system; EEG, electroencephalogram.

a Number of subjects experiencing an AE which were assessed by the Site Investigator as possibly, probably or definitely related to study drug during the trial period. One placebo subject (#601) who was withdrawn on day 8 owing to abnormal EEG and one b.i.d. subject (#607) who withdrew consent on dy 1 for personal reasons are excluded from this table but are included in the Supplementary Table S1.

**Table 3:** Baseline biomarker levels and the probability of response to NSI-189 at day 28

*Pt. ID*	*Designation*	*Probablilty*
501	PR	1.000
502	R	1.000
504	R	1.000
505	R	1.000
506	R	1.000
508	NR	0.032
602	R	1.000
603	R	0.961
604	PR	1.000
606	PR	0.998
608*	PR*	0.000*
627	R	0.980
701	R	1.000
702	R	0.987
703	R	0.000
705	NR	0.031
706	R	1.000
707	R	0.995

Abbreviations: A1AT, alpha-1-antitrypsin; BNF, brain-derived neurotrophic factor; EGF, epidermal growth factor; MADRS, montgomery-asberg depression scale; MPO, myeloperoxidase; NR, non-responder; PR, partial responder; R, responder; TNFR2, tumor necrosis factor receptor type 2.

Indicates the individual patient identifier, and the response group designation (R, PR and NR) and the predicted probability of response based upon analysis of baseline biomarker data. We used partial least squares discriminant analysis to choose the important variables (BDNF, EGF, MPO, TNFR2 and A1AT) in predicting response. One possible outlier (*) was patient 608 who was designated a partial responder based on a single MADRS score of 16 which indicated a change from moderate to mild depression. The observed probability was consistent with the patient being a NR.

## Notes

Dr Johe is an employee of Neuralstem. The remainder of the authors have received research support from Neuralstem. For a detailed list of lifetime disclosures of Dr. Fava, please see http://mghcme.org/faculty/faculty-detail/maurizio_fava
