# Erratum: A Phase 1B, randomized, double blind, placebo controlled, multiple-dose escalation study of NSI-189 phosphate, a neurogenic compound, in depressed patients

**DOI:** 10.1038/mp.2016.140

**Published:** 2016-08-16

**Authors:** M Fava, K Johe, L Ereshefsky, L G Gertsik, B A English, J A Bilello, L M Thurmond, J Johnstone, B C Dickerson, N Makris, B B Hoeppner, M Flynn, D Mischoulon, G Kinrys, M P Freeman

**Keywords:** Clinical pharmacology, Randomized controlled trials, Depression, Drug therapy

**Correction to:** *Molecular Psychiatry* advance online publication, 8 December 2015; doi:10.1038/mp.2015.178

Following publication of this paper, the authors noticed that Figures 5a and b contained the same image. In addition, there was a typo in Table 3 at #703. The correct figure and table appear here.

**Figure 5:**
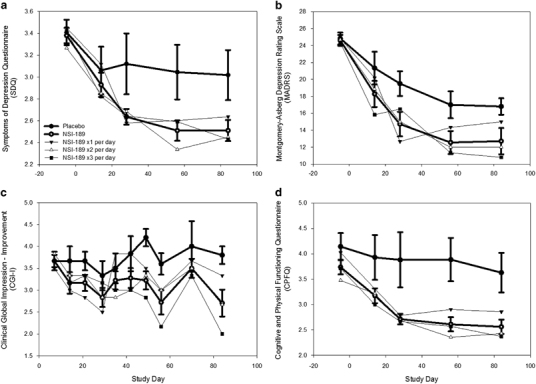
(**a**–**d**) Efficacy outcomes in means over time. PowerPoint slide

**Table 3:** Baseline biomarker levels and the probability of response to NSI-189 at day 28

*Patient. ID*	*Designation*	*Probablilty*
501	PR	1.000
502	R	1.000
504	R	1.000
505	R	1.000
506	R	1.000
508	NR	0.032
602	R	1.000
603	R	0.961
604	PR	1.000
606	PR	0.998
608*	PR*	0.000*
627	R	0.980
701	R	1.000
702	R	0.987
703	NR	0.000
705	NR	0.031
706	R	1.000
707	R	0.995

Abbreviations: A1AT, alpha-1-antitrypsin; BNF, brain-derived neurotrophic factor; EGF, epidermal growth factor; MADRS, montgomery-asberg depression scale; MPO, myeloperoxidase; NR, non-responder; PR, partial responder; R, responder; TNFR2, tumor necrosis factor receptor type 2. Indicates the individual patient identifier, and the response group designation (R, PR and NR) and the predicted probability of response based upon analysis of baseline biomarker data. We used partial least squares discriminant analysis to choose the important variables (BDNF, EGF, MPO, TNFR2 and A1AT) in predicting response. One possible outlier (*) was patient 608 who was designated a partial responder based on a single MADRS score of 16 which indicated a change from moderate to mild depression. The observed probability was consistent with the patient being a NR.

## PowerPoint slides

PowerPoint slide for Fig. 1

