# Supplementary material for: A Phase 1B, randomized, double blind, placebo controlled, multiple-dose escalation study of NSI-189 phosphate, a neurogenic compound, in depressed patients
Source: Mol Psychiatry. 2015 Dec 8;21(10):1372–80. doi: 10.1038/mp.2015.178 (PMC5030464; doi:10.1038/mp.2015.178)
Supplement: Supplementary Information [file mp2015178x1.docx]

Supplemental Table 1: Adverse Events *

|  | |  |  |  |  |  |  |
| --- | --- | --- | --- | --- | --- | --- | --- |
|  |  |  |  |  |  |  |  |
| Side Effect | | Placebo | 40 mg QD | 40 mg BID | 40 mg TID | Pooled  Active | Pooled Placebo |
|  |  | (n=6) | (n=6) | (n=6) | (n=6) | (n=18) | (n=6) |
| Autonomic | |  |  |  |  |  |  |
|  | Dry mouth | 0 | 0 | 2 | 0 | 2 (11.1%) | - |
|  | Palpitation | 0 | 1 | 0 | 0 | 1 (5.6%) | - |
| CNS/Psychiatric | |  |  |  |  |  |  |
|  | Headache | 3 | 3 | 3 | 3 | 9 (50%) | 3 (50%) |
|  | Dizziness | 1 | 0 | 1 | 4 | 5 (27.8%) | 1 (5.6%) |
|  | Somnolence  Fatigue  Restlessness | 1  0  0 | 3  1  0 | 1  0  0 | 1  0  1 | 5 (27.8%)  1 (5.6%)  1 (5.6%) | 1 (5.6%)  -  - |
|  | Poor Quality of Sleep | 0 | 1 | 0 | 0 | 1 (5.6%) | - |
|  | Nightmare / vivid dream | 0 | 1 | 1 | 1 | 3 (16.7%) | - |
|  | Paresthesia | 0 | 1 | 0 | 1 | 2 (11.1%) | - |
|  | Insomnia | 0 | 1 | 1 | 1 | 3 (16.7%) | - |
|  | Irritability | 0 | 1 | 0 | 0 | 1 (5.6%) | - |
|  | Difficulty concentrating | 1 | 0 | 0 | 0 | - | 1 (5.6%) |
|  | Hyperthymia | 1 | 0 | 0 | 0 | - | 1 (5.6%) |
| Gastrointestinal | |  |  |  |  |  |  |
|  | Dyspepsia | 1 | 0 | 0 | 0 | - | 1 (5.6%) |
|  | Abdominal pain | 1 | 0 | 0 | 0 | - | 1 (5.6%) |
|  | Nausea | 0 | 0 | 0 | 2 | 2 (11.1%) | - |
|  | Skin and Subcutaneous Tissue Disorders | | |  |  |  |  |
|  | Skin Pain | 0 | 1 | 0 | 0 | 1 (5.6%) | - |
|  | Rash | 0 | 0 | 0 | 1 | 1 (5.6%) | - |
| Note: * Number of subjects experiencing an AE which were assessed by the Site Investigator as possibly, probably or definitely related to study drug during the trial period. | | | | | |  |  |

|  | | Placebo | 40 mg QD | 40 mg BID | 40 mg TID |
| --- | --- | --- | --- | --- | --- |
|  |  | (n=6) | (n=6) | (n=6) | (n=6) |
|  | |  |  |  |  |
|  | Individuals with any AE | 5 (83.3%) | 6 (100%) | 4 (66.7%) | 6 (100%) |
|  |  |  |  |  |  |
|  | |  |  |  |  |
|  |  |  |  |  |  |
|  |  |  |  |  |  |
